# Ipsilateral access portal venous embolization (PVE) for preoperative hypertrophy exhibits low complication rates in Clavien-Dindo and CIRSE scales

**DOI:** 10.1186/s42155-021-00227-5

**Published:** 2021-05-17

**Authors:** Roland Brüning, Martin Schneider, Michel Tiede, Peter Wohlmuth, Gregor Stavrou, Thomas von Hahn, Andrea Ehrenfeld, Tim Reese, Georgios Makridis, Axel Stang, Karl J. Oldhafer

**Affiliations:** 1 Radiology and Neuroradiology, Asklepios Hospital Barmbek, Ruebenkamp 220, 22307 Hamburg, Germany; 2 Faculty of medicine, Bavariaring 19, 80336 München, Germany; 3 Biostatistics, ProResearch, Lohmuehlenstrasse 5, 20099 Hamburg, Germany; 4 Department of General, Visceral and Thoracic Surgery, Surgical Oncology, Klinikum Saarbruecken, Winterberg 1, 66199 Saarbrücken, Germany; 5 Gastroenterology, Hepatology and interventional Endoscopy, Asklepios Hospital Barmbek, Ruebenkamp 220, 22307 Hamburg, Germany; 6 Medical Faculty, Semmelweis University Budapest, Üllői út 26, 1085 Budapest, Hungary; 7 Department of Surgery, Division of Liver-, Bileduct- and Pancreatic Surgery, Asklepios Hospital Barmbek, Ruebenkamp 220, 22307 Hamburg, Germany; 8 Oncology, Asklepios Hospital Barmbek, Ruebenkamp 220, 22307 Hamburg, Germany

**Keywords:** Portal vein embolization, PVE, Portal vein occlusion, FLR, Hypertrophy

## Abstract

**Background:**

Portal venous embolization (PVE) is a minimal invasive preoperative strategy that aims to increase future liver remnant (FLR) in order to facilitate extended hemihepatectomy. We analyzed our data retrospectively regarding complications and degree of hypertrophy (DH).

Methods: 88 patients received PVE either by particles / coils (*n* = 77) or by glue / oil (*n* = 11), supported by 7 right hepatic vein embolizations (HVE) by coils or occluders. All complications were categorized by the Clavien- Dindo (CD) and the CIRSE classification.

**Results:**

In 88 patients (median age 68 years) there was one intervention with a biliary leak and subsequent drainage (complication grade 3 CD, CIRSE 3), two with prolonged hospital stay (grade 2 CD, grade 3 CIRSE) and 13 complications grade 1 CD, but no complications of grade 4 or higher neither in Clavien- Dindo nor in CIRSE classification. The median relative increase in FLR was 47% (SD 35%). The mean pre-intervention standardized FLR rose from 23% (SD 10%) to a post-intervention standardized FLR of 32% (SD 12%). The degree of hypertrophy (DH) was 9,3% (SD 5,2%) and the kinetic growth rate (KGR) per week was 2,06 (SD 1,84).

**Conclusion:**

PVE and, if necessary, additional sequential HVE were safe procedures with a low rate of complications and facilitated sufficient preoperative hypertrophy of the future liver remnant.

## Background

Recent advances in hepatobiliary surgery and the possibility of safely removing larger portions of the liver have improved the proportion of potentially resectable tumors in malignant liver disease (Abulkhir et al. 2008). However, if the proportion of the anticipated liver volume that remains in situ after surgery (the future liver remnant (FLR)) is small, patients remain at risk for developing postoperative complications such as liver failure (Abulkhir et al. 2008; Azoulay et al. 2000). The FLR is measured before planning surgery, as its volume has been shown to be a predictor for postoperative liver dysfunction (Shoup et al. 2003; Ribero et al. 2007). If necessary, a small FLR can be enlarged by hypertrophy strategies. The two most established methods are Associating Liver Partition with Portal vein ligation for Staged hepatectomy (ALPPS) (Schnitzbauer et al. 2012) and Portal Venous Embolization (PVE) (Makuuchi et al. 1990; de Baere et al. 1993; Abdalla et al. 2002), both of which are techniques to redirect portal blood flow, in an attempt to promote hypertrophy of nonembolized /nontreated segments that will remain after resection (Madoff et al. 2016).

The initial reports of the percutaneous approach to portal vein branches occlusion date were initiated, when Takayasu et al. reported on contralateral growth following ipsilateral (tumor) obstruction (Takayasu et al. 1986) and Kinoshita et al. (Kinoshita et al. 1985) reported on the transcutaneous transhepatic approach. Shortly after, animal studies exhibited the ability to regenerate (Tanaka et al. 1994) in a two-stage concept. Since then, PVE was developed and has gained relevant support worldwide (Luz et al. 2020).

An increased FLR volume once archived helps patients previously considered ineligible for resection (Abulkhir et al. 2008; Azoulay et al. 2000; Madoff et al. 2005). There is consensus that the necessary future liver volume is in the range of 25–40% and mainly depends on the quality of liver tissue (Abdalla et al. 2002; Benson 3rd et al. 2013; Kubota et al. 1997; Shirabe et al. 1999; de Meijer et al. 2010), and therefore on the underlying disease and histology.

From a technical standpoint, PVE can be varied substantially. We used two different embolization materials: Coils and particles versus glue and oil. An additional intervention using a different approach (hepatic vein embolization (HVE)) was employed in cases with a hypertrophy not sufficient for the next surgical step. In this retrospective evaluation, we sought to evaluate the degrees of hypertrophy, the kinetic growth of the future liver remnant and complications with respect to PVE procedures. Based on a preliminary analysis of our data, our hypothesis was that independent of materials used, PVE would allow a safe procedure with only few and low grade complications.

## Materials and methods

### Patients

In a retrospective analysis of our hospital’s data base, a total of 114 interventions were identified between 2013 and 2019.

Inclusion criteria were primary or secondary liver lesions planned for extended right hemihepatectomy (ERH). Disease had to be liver dominant, and a tumor board vote had to be positive towards a hypertrophy model and hemihepatectomy. Pre- and postinterventional CT scans, laboratory parameters and biometric data needed to be available to be included.

The following patients were excluded from further analysis: 15 patients were lost to follow up or had insufficient scan quality, in 2 patients the retrospective data of the intervention / lab data were incomplete, and in another 2 patients there was an atypical (left) approach.

Ninety-five interventions in 88 patients were included and further analyzed: 88 PVE were evaluated and 7 HVE interventions were used. The patients and their hypertrophy data were also grouped by the underlying disease, please refer to Table 1 for further patient details.

**Table 1:** Patient data

	Mean	SD
Total patients included^a^	88	
Age (years; at the time of procedure) (lower quartile, median, upper quartile)	59/68/74	11
Gender male	60	
Weight (kg)	77	16
Height (cm)	173,4	7,7
BMI (lower quartile, median, upper quartile)	24,7/27,5/31,1	
BSA (Mosteller) (m2)	1,90	0,27
TELV (cm2)	1635	269
Material and methods used		
PVE with Coils/PVA	77	
PVE with Glue/Lipiodol	11	
HVE with coils/occluder^b^	7	
Malignancies		
Colorectal liver metastasis (CRLM)^c^	43	
Central bile duct/ Cholangiocellular carcinoma (CCC)^d^	27	
Hepatocellular carcinoma^e^	8	
Other^f^	10	

^a^Inclusion criteria were primary or secondary liver lesions planned for extended right hemihepatectomy (ERH). Disease had to be liver dominant, and a tumor board vote had to be positive towards a hypertrophy model and hemihepatectomy. Pre- and postinterventional CT scans, laboratory parameters and biometric data needed to be available to be included. Ninety-five interventions in 88 patients were analyzed. The following patients were excluded from further analysis: 15 patients were lost to follow up or had insufficient scan quality, in 2 patients the retrospective data of the intervention / lab data were incomplete, and in another 2 patients there was an atypical (left) approach. ^b^One HVE was perfomed within the same session as PVE

^c^Of the patients with CRLM, 25 had previous surgery (as did all CRLM HVE patients), 3 had radiofrequency ablation (RFA), and 6 patients received FOLFOX or Bevacicumab (Avastin®)

^d^Of the patients with central bile duct tumors (intrahepatic CCC and or perihilar cholangiocarcinomas (Klatskin)), 5 had prior surgery and 5 had other previous interventions (such as ERCP or PTCD) - in total there were 9 pre-treated patients in this group

^e^Regarding the patients with HCC, one patient had a previous trans-arterial chemoembolization and none had previous surgery

^f^Regarding the patients with other malignancies, 3 had previous surgery, 3 and 2 had other pretreatments (Y90 and RFA)

### Intervention

PVE was performed under general anesthesia by an experienced interventionalist (> 10 years of interventional radiology) and we used an ipsilateral approach to prevent damage to the FLR. The ultrasound-guided transcutaneous transhepatic approach with a dedicated puncture device (Toshiba Aplio XV; Toshiba Europe GmbH, Neuss, Germany) was performed in cooperation with an experienced sonographer in order to minimize punctation events. Following successful puncture of a peripheral portal branch, access was secured by a 6 French sheath. Next, a 4 French pigtail catheter (Cook Medical, Bloomington, IN) was introduced into the central portal vein, and a direct portography in three planes was performed (typically 0, − 25 und + 25 degrees rotation). Then, a 5 or 6 French S1 Sidewinder Catheter (Cook Medical, see above) was routinely introduced to selectively access branches of the right portal vein; and used for embolization. We did not use microcatheters on a routine basis. The selection of the embolization material in each case was to the discretion of the responsible interventionalist.

In the coils and particles group, PVA particles (Contour™ PVA Particles, 250–355 mm; Terumo, Tokyo, Japan) and fibred coil embolization were used (mREYE® spiral sizes 3–12 mm, Cook Medical, see above). Because of individual anatomical conditions, Amplatzer™ Vascular Plugs (St Jude Medical, Saint Paul, MN, United States) were used in 2 PVE patients. The embolization procedure in the glue / oil group was performed using a mixture of Lipiodol® (Guerbet, France) and synthetic surgical glue (Gluebran®, GEM Viareggio Italy); usually diluted 3:1. Segment 4 branches arising from the left branch of the portal vein were selectively embolized whenever possible. At the end of the procedure, complete occlusion of target vessels was documented by portography.

A right hepatic vein embolization was performed by transjugular access and embolization and occlusion of the right hepatic vein using mReye Coils, Amplatz occluders or a combination of both. This subgroup was analyzed separately on a new baseline before the second intervention.

Complications arising from all procedures were drawn from the individual records during the hospital stay, from both angiographic reports and discharge letters, and were categorized using the Clavien- Dindo classification (Dindo et al. 2004) and using the CIRSE classification (Filippiadis et al. 2017) for intervention-related complications.

Routinely, a follow-up CT was performed 5 weeks after PVE. Follow-up imaging and volumetry was CT-based on multi-slice scanners [usually on a GE Optima 660 (GE Healthcare, Pittsburgh, PA) or a Philips Brilliance 128 (Philips Healthcare, Best, Netherlands)], 5 mm reconstructions were carried out in three planes. All volumetric post-processing was carried out on an Advantage Workstation 4.1.2 (GE Healthcare, see above), by manually outlining Segments 2 and 3, using threshold values. Total estimated liver volume (TELV) was calculated using the formula published by Vauthey et al. (Vauthey et al. 2000), based on the body surface area (BSA) by Mosteller. Parameters such as relative growth (%), DH, and KGR were calculated, as described by Shindoh (Shindoh et al. 2013) in order to assess both hypertrophy at the time of control and the dynamic development.

In selected cases HVE was added as a second procedure after interdisciplinary case discussion, when left lobe hypertrophy after PVE was insufficient for surgery (sFLR below 30). In total seven HVE were performed, of those were 6 in the described subsequent second step; the 7th HVE was performed at the discretion if the interventionalist in a PVE session in a 57 year old female with CRLM: two hepatic vein occluders assisted the portal embolization, as the coil and particle based PVE technically seemed not sufficient.

### Statistical methods

Continuous data were summarized as means +/− standard deviations or as medians [25th and 75th percentiles] as appropriate. Categorical data were presented as N (%). Outcome data (FLR, KGR) were shown by follow up time and intervention methods using scatterplots and a smoothed local regression curve. Response and histology were presented with boxplots. All calculations were performed with the statistical analysis software R.

## Results

In the entire group hypertrophy of Segments 2 and 3 reached a median relative increase was 47% (SD 35%). The mean pre-intervention sFLR rose from 23% (SD 10%) to a post-intervention sFLR of 32% (SD 12%). The DH was 9,3% (SD 5,2%); and the KGR was 2,06 (SD 1,84). The FLRBWR (FLR to BW ratio) was 0,69 (SD 0,26).

**Fig. 1:**
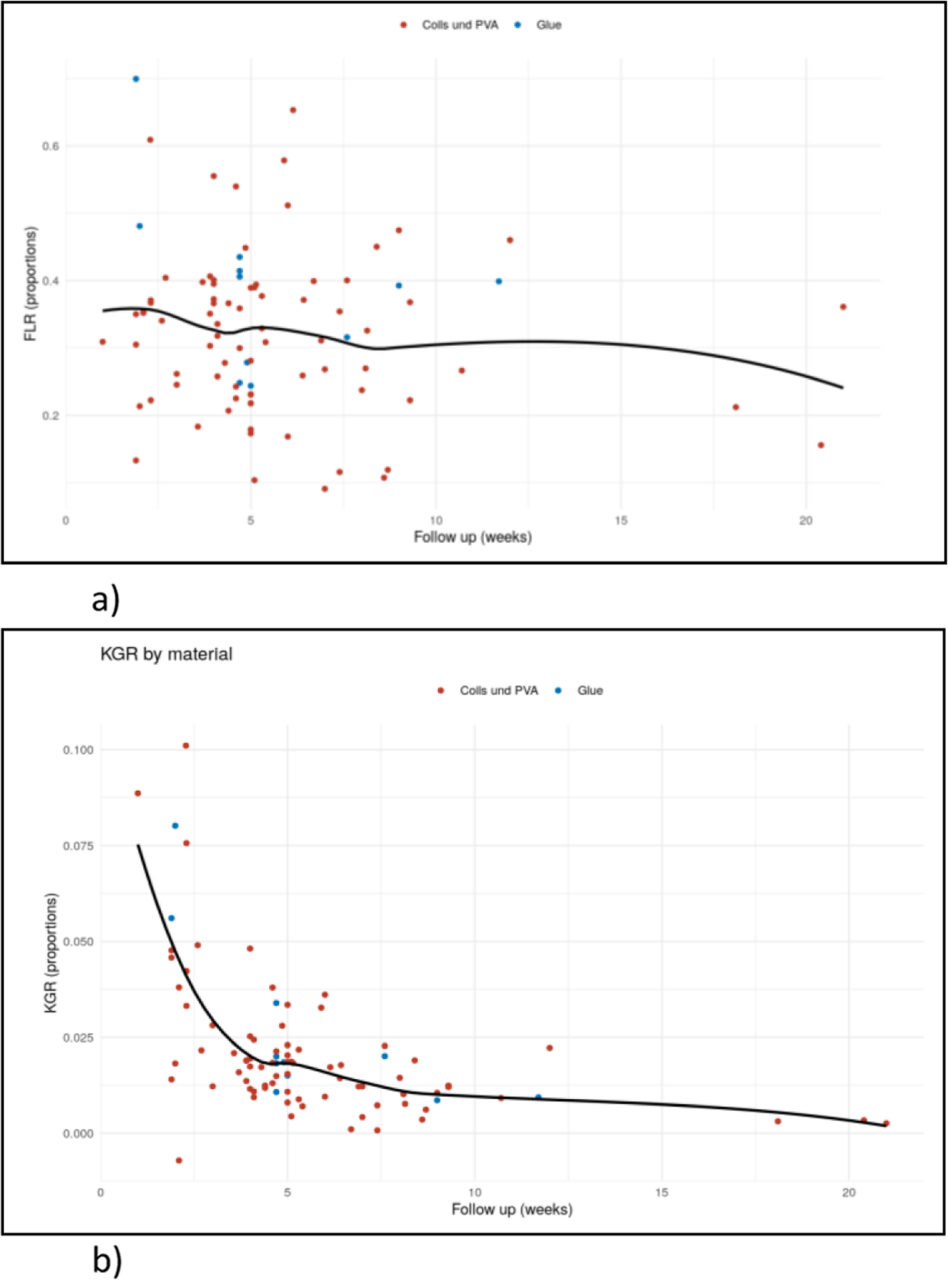
**a**, **b**: In the entire group, the relative growth of FLR using PVA particles and coils was 44% ± 28%; using glue and HVE it was 43% ± 23% and 87% ± 77%, respectively. **a**: Proportional FLR by methods (coils and PVA versus Glue) and follow up time (weeks). Each point displays the follow up measurement of one patient. The proportional growth of post-intervention sFLR using PVA/coils was 31,9% ±11,9%, and using glue and HVE, was 39,2% ±12,9% and 27,5% ±5,7%, respectively. The black line represents a smoothed local regression curve (across methods). **b**: Proportional KGR by methods (coils and PVA versus Glue) and follow up time (weeks). Each point displays the follow up measurement of one patient. The KGR using PVA/coils was 2.04% per week ±1.82%, using glue was 2.64% ± 2.24% per week and using HVE was 1.32% ± 1.17% per week. The black line represents a smoothed local regression curve (across methods)

Details of the effectiveness and grade of hypertrophy of FLR by the different embolization methods over time are given in Fig. 1a and details of the KGR are given in Fig. 1b.

**Fig. 2:**
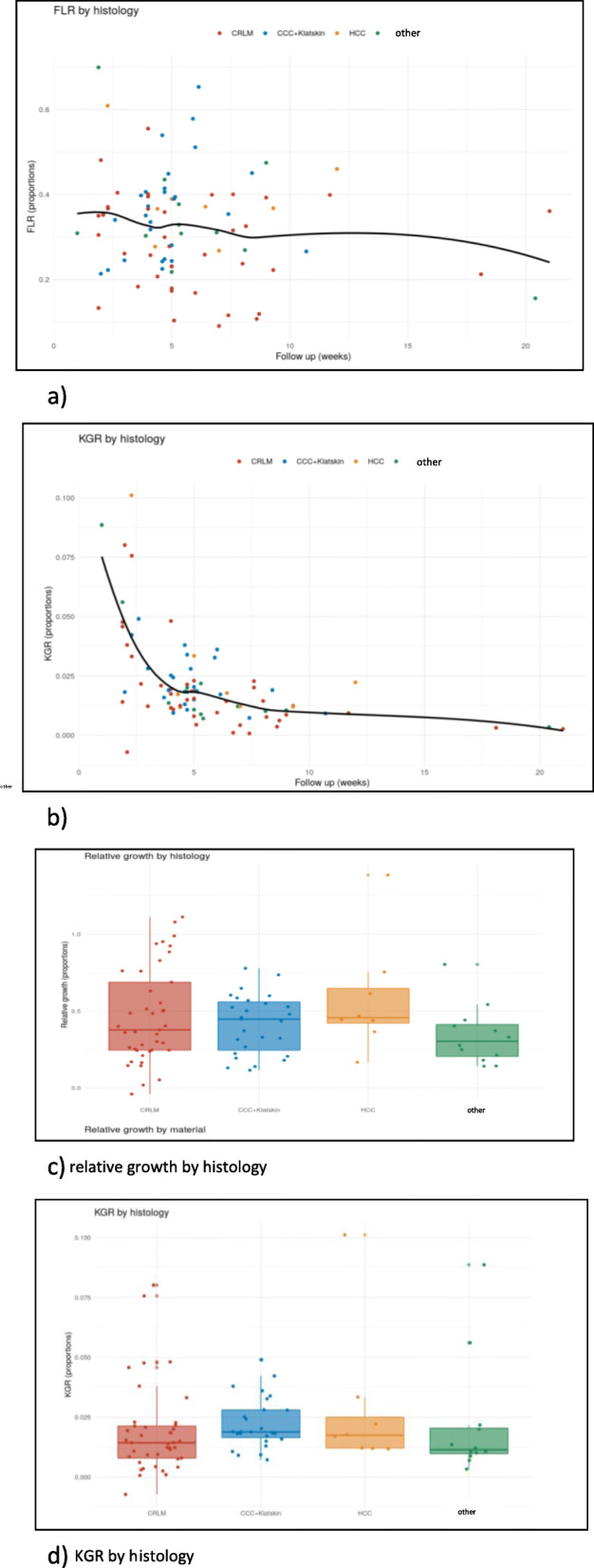
Hypertrophy analysis by different histologies: **a**: Proportional FLR by histology and follow up time (weeks). Each point displays the follow up measurement of one patient. The black line represents a smoothed local regression curve (across histologies). **b**: Proportional KGR by histology and follow up time (weeks). Each point displays the follow up measurement of one patient. The black line represents a smoothed local regression curve (across histologies). **c** and **d**: The relative growth and the KGR are given in box- plots: In our subgroup analysis we found a median relative growth of the FLR in the CRLM group of 52% (SD 43%), in the group of central bile duct tumors of 37% (SD 18%) and in the HCC group of 58% (SD 37%). **c** relative growth by histology. **d** KGR by histology

The subgroup analysis of effectiveness and grade of hypertrophy of FLR by histology is displayed in Fig. 2a and b. To visualize the differences between the relative growth of group of malignancy, these results are given in Box-plots in Fig. 2c and d.

The following complications occurred: There was one PVE intervention necessitating subsequent drainage and revision of the central stents in a 52 year old female suffering from central bile duct tumor with a subsequent bilioma which had to be addressed by CT-guided drainage (Clavien- Dindo grade 3, CIRSE grade 3), however with sufficient hypertrophy and extended right hemihepatectomy to follow. Another PVE intervention had prolonged intermediate care in a 72 year old female a central portal vein stasis despite injection of 5000 IE heparin, this finding however was not reproduced in subsequent US and CT scans, so another PVE session completed the procedure (Clavien- Dindo grade 2; CIRSE 3), and the third CIRSE 3 complication was a 69 year old female with self limiting hematemesis. There were 11 minor complications Grade 1 Clavien- Dindo. Of those interventions with a complication Clavien- Dindo grade 1–3, seven (54%) received treatment before PVE such as radiofrequency ablation. Two of those interventions (15%) were performed using glue and the others using coils/PVA, reflecting almost a normal distribution in our cohort. During four treatments displaced mReye coils were subsequently retrieved in the same session by a Snare® device (GooseNeck® Snare Kit; Covidien eV3, Plymouth, MN) (CIRSE grade1). Hospital stay was prolonged < 48 h in 6 patients (CIRSE grade 2) and > 48 h in 3 patients (CIRSE grade 3). For further details please refer to Table 2.

**Table 2:** Complications of PVE scored by the Clavien Dindo score and the CIRSE score

Complications Dindo-Clavien	Numbers (percentage)	Complications CIRSE grading	Numbers (percentage)
Grade 1 (Any deviation from the normal postoperative course without the need for pharmacological treatment or surgical, endoscopic and radiological interventions)	11/88 (12,5%)	Grade 1 (Complication during the procedure which could be solved within the same session; no additional therapy, no postprocedure sequelae, ...)	4/88 (4,5%)
Grade 2 (Requiring pharmacological treatment with drugs other than such allowed for grade I complications)	1/88 (1,1%)	Grade 2 (Prolonged observation including overnight stay (as a deviation from the normal post-therapeutic course\48 h)	6/88 (6,8%)
Grade 3 (Requiring surgical, endoscopic or radiological intervention)	1/88 (1,1%)	Grade 3 (Additional postprocedure therapy or prolonged hospital stay (48 h) required; no postprocedure sequelae)	3/88 (3,4%)
Grade 4 (Life-threatening complication (including CNS complications)* requiring IC/ICU-management)	0 (0%)	Grade 4 (Complication causing a permanent mild sequelae (resuming work and independent living))	0 (0%)
Grade 5 (death)	0 (0%)	Grade 5 (Complication causing a permanent severe sequelae (requiring ongoing assistance in daily life))	0 (0%)
		Grade 6 (death)	0 (0%)

PVE were followed by ERH in 61/88 patients (69%) across subgroups. In 6 cases, to enable more profound hypertrophy, HVE was added as a second procedure after interdisciplinary case discussion. In all 6 cases the left lobe hypertrophy after PVE was insufficient for surgery (mean sFLR 26,6). After PVE and additional HVE 4 out of 6 patients showed hypertrophy results that enabled them to receive the aimed hemihepatectomy.

## Discussion

The most striking finding in our analysis was the low number of severe complications – those exceeding Clavien- Dindo grade 1 were about 2,2% and none exceeding grade 3. We assume that the right- sided approach under expert ultrasound guidance and the careful use of the embolization material could have contributed to this low complication rate: it has the benefit of avoiding damage to the FLR at the cost of more difficult navigation using reverse angle catheters, as described by Madoff et al. (Madoff et al. 2016). Among the available scores, we selected to grade according to the most established (and published) Clavien- Dindo classification, however, this classification is intended for major surgery, and the CIRSE scale for interventions (Filippiadis et al. 2017).

Other more aggressive embolization materials exhibited high growth rates, but PVE with N-butyl cyanoacrylate exhibited major complications in 19 patients (3.13%), while minor complications occurred in 38 patients (6.26%) (Wajswol et al. 2018). The use of ethanol- based PVE exhibited 9 major complications out of 151 (6%) PVE sessions and minor complications such as fever in 47 of 151 (31.1%) of procedures (Sakuhara et al. 2012). Morbidity has also been reported in two elderly patients with Klatskin tumors following PVE with gelfoam and coils and subsequent sepsis (Lee et al. 2017). Using the combined approach of HVE/PVE in the small group of 10 patients, two grade 3 and one grade 3 Clavien- Dindo complications have been reported (30%) (Guiu et al. 2017). Conversely, classic PVE-related complications exceeding CD grade 2 were found in only 0.6% of the 494 patients of a Japanese series (Ebata et al. 2012). Summing up all different approaches, an early meta-analysis of Abulkir et. Al. reported a historical overall morbidity for PVE of 2.2% (Abulkhir et al. 2008).

The low number of complications in our series with the endocascular and transcutaneous approach also compare favorably to the surgical technique of ALPPS, where complications leading to mortality are reported ranging from 4.2% to 29.1% of patients (Linecker et al. 2017; Wanis et al. 2021). Another group reported severe morbidity (Clavien- Dindo ≥3a) to occur in 50% (3/6) of the rescue ALPPS, 71% (10/14) of the ALPPS, and 38% (13/34) of the TSH/PVE patients (*p* = 0.300) (Narita et al. 2011). Consequently, for example Hahn et al., (Hahn et al. 2019) modified their surgical strategy and reported a Clavien-Dindo III-IV morbidity of 22% and mortality of 0%.

In our evaluation of hypertrophy following PVE we found a median relative increase of FLR of 47% across the entire group, which compares equally or favorably with previous publications, as various groups have previously reported a relative hypertrophy in a range of 30% - 50% (Wajswol et al. 2018; Geisel et al. 2014; van Lienden et al. 2013; Zeile et al. 2016).

Hypertrophy varies substantially between technical approaches: our subgroup analysis found the relative growth of FLR almost similar between coils and glue (44% and 43%, respectively). Conversely, there have been a publications indicating that glue yields a better result, as the utilization of N-butyl cyanoacrylate has been reported by Wajswol et al. to increase FLR by 49.4% ± 1.3% (Wajswol et al. 2018). Jaberi et al. reported a DH of 16.2% using glue vs 12.3% using PVA or combined with Amplatzer (*p* < .009) (Jaberi et al. 2016). On the other hand, hypertrophy was described using PVA alone by Geisel et al. at 30.9% and in combination with coils at 53.3% (Geisel et al. 2014). However, differences between reported growth rates were huge and may have been a result of different patient selection and the presence or absence of concurrent medication – limiting comparability. This situation with a huge span of DH was outlined in the recent review by Luz et al. (Luz et al. 2020). The review of van Lienden et.al., (van Lienden et al. 2013) calculated a mean DH to be 37.9%.

Trying to find a predictor for postsurgical failure we shared a special interest in KGR (defined as the degree of hypertrophy at initial postPVE volume assessment divided by number of weeks elapsed after PVE). It has been shown by Shindoh et al. (Shindoh et al. 2013), that the volumetric sFLR threshold for safe resection of 30% was not significantly associated with static hypertrophy. As a consequence, the same group presented the kinetic growth rate to be the more accurate predictor for postsurgical failure since the kinetic growth rate (KGR) has been suggested to better reflect the regenerative ability of the liver in patients undergoing PVE as it takes into account the duration necessary to achieve an adequate volume Liver kinetic growth rate predicts postoperative liver failure after ALPPS (Kambakamba et al. 2016).

The KGR we found in our glue group was 2,64% per week and in the coils group 2,04% per week. A KGR of 3,5% per week in the glue group and 2,6% per week in the PVA group were reported by Jaberi (Jaberi et al. 2016). However, Guiu et al. reported in a smaller cohort a very high kinetic growth rate of 53% in 7 days following their combined PVE and HVE approach (Guiu et al. 2017), however, at the cost of increased sequelae as shown above.

To date, important factors affecting hypertrophy remain uncertain and may be based on patient selection, to various methods of embolization and so on. A previous analysis of cofactors outlined negative predictive factors such as the formation of porto-portal collaterals (*p* = 0.004) (Geisel et al. 2014).

Regarding the effectiveness of hypertrophy between histological groups in the subgroup of colorectal liver metastasis (CRLM) we found a median relative growth of the FLR of 52%. However, there was a remarkable range of growth with a standard deviation of 43%, which we assume to be linked to the frequent pre-treatment in this group. The effects of pretreatment have been reported previously, although the effect of chemotherapy is not clear (Madoff et al. 2016). Zorzi et al. (Zorzi et al. 2008) reviewed FLR hypertrophy after PVE in patients with CRLM and showed comparable degrees of hypertrophy of treated patients when compared with the no chemotherapy group 4 weeks after PVE. However, in a small series of 15 consecutive patients by Beal et al. (Beal et al. 2006) the increase in FLR volume was reduced in the setting of chemotherapy (*p* < .016).

In the group of central bile duct tumors there was a median relative growth of the FLR of 37% (SD 18%). Nine of 27 patients received pre-treatment, of those, drainage of dilated bile ducts (*n* = 5). A DH of more than 20% has been reported in the large cohort of Nagino et al. (Nagino et al. 2006) to a pre-operative FLR of 460 cm^3^ by Ebata (Ebata et al. 2012).

In the HCC group we found a relative growth of the FLR of 58% (SD 37%). This DH exceeds the average result of 31% increase after PVE as reported in the recent review of Tustumi and coworkers (Tustumi et al. 2018). Interestingly, we did not observe a lack of growth by either cirrhosis or hepatic steatosis, typically preceding the development of HCC. This is in accordance with the report of Deipolyi et al. (Deipolyi et al. 2017) who did not observe neither effect in their subgroup.

The PVE based hypertrophy concept in our own group resulted in a rate of curative intended surgery of 69%. Intra- or extrahepatic disease progression in the interval was the main reason to preclude these approaches. Another reason was general medical deterioration, as was previously mentioned by Deipolyi (Deipolyi et al. 2017). The meta-analysis of van Lienden et al. (van Lienden et al. 2013) has outlined 6.1% local intrahepatic tumor progression in the FLR; and 8.1% extrahepatic tumor spread. Wajswol et al. (Wajswol et al. 2018) reported that 76% of PVE candidates underwent surgical resection. A 20% drop-out rate after first stage resection from disease progression was reported in another two-stage hepatectomy series (Narita et al. 2011; Hahn et al. 2019). In central bile duct tumors, a 25% rate of non-resectability has been reported in a larger series by Ebata et al. (Ebata et al. 2012).

If growth of the FLR did not match the anticipated values, six patients underwent a subsequent session addressed by a right hepatic vein embolization (HVE). Following this sequential HVE, 4 of these patients could then receive major surgery. A combination of PVE and HVE in the same session was reported by Le Roy and coworkers with a DH of 52.6% (Le Roy et al. 2017). Guiu et al. (Guiu et al. 2017) found the FLR increased by 64.3% in a similar combined approach at day 21. On the other hand, Hwang et al. (Hwang et al. 2015) reported 42 patients to have a documented DH of FLR of 28.9% after sequential PVE-HVE.

Limitations of our study included, that our data were analyzed in a retrospective fashion and were single center based including a learning curve, so there will be limitations on their impact. There was no uniformity in embolization technique and there was heterogeneity of the underlying liver tumors. However, there are no multicenter prospective studies either comparing FLR hypertrophy between ALPPS and PVE or regarding different embolization materials. Our evaluation did not include local functional capacity measurable e.g. by SPECT (not possible in our center). The measurement of indocyanine retention ratios were not available at that time and thus was not part of our analysis.

In conclusion PVE were safe procedures with a low rate of complications that allows preoperative hypertrophy and kinetic growth of the future liver remnant.

## Data Availability

The datasets generated during the current study will be available in the open data repository at lmu-munich.de. A DOI will be created upon acceptance of the manuscript.

## Abbreviations

FLR: Future liver remnant
DH: Degree of hypertrophy (=sFLR after PVE – sFLR befove PVE)
FLRBWR: Future liver remnant to body weight ratio
HVE: Hepatic vein embolization
PVE: Portal vein embolization
sFLR: Future liver remant standardized to the BMI by Mosteller, according to the formula sFLR% = ((FLR (cm3)/TELV (cm3))*100
BMI: Body mass index
KGR: Kinetic growth rate defined as the degree of hypertrophy at initial postPVE volume assessment divided by number of weeks elapsed after PVE, according to the formula KGR = degree of hypertrophy/weeks
PVA: Polyvinyl alcohol particles
HCC: Hepatocellular carcinoma
CRLM: Colorectal liver metastasis
SD: Standard deviation
TELV: Total estimated liver volume, according to the formula, according to the formula TELV (cm3) = -794,41 + 1267,28 * BSA
ERH: Extended right hemihepatectomy
ALPPS: Associating Liver Partition with Portal vein ligation for Staged hepatectomy
CD: Clavien- Dindo classification
CIRSE classification of complications: Classification of complications

## References

[CR1] Abdalla EK, Barnett CC, Doherty D, Curley SA, Vauthey JN (2002). Extended hepatectomy in patients with hepatobiliary malignancies with and without preoperative portal vein embolization. Arch Surg (Chicago, Ill. : 1960).

[CR2] Abulkhir A, Limongelli P, Healey AJ, Damrah O, Tait P, Jackson J, Habib N, Jiao LR (2008). Preoperative portal vein embolization for major liver resection: a meta-analysis. Ann Surg.

[CR3] Azoulay D, Castaing D, Krissat J, Smail A, Marin Hargreaves G, Lemoine A, Emile JF, Bismuth H (2000). Percutaneous portal vein embolization increases the feasibility and safety of major liver resection for hepatocellular carcinoma in injured liver. Ann Surg.

[CR4] Beal IK, Anthony S, Papadopoulou A, Hutchins R, Fusai G, Begent R, Davies N, Tibballs J, Davidson B (2006). Portal vein embolisation prior to hepatic resection for colorectal liver metastases and the effects of periprocedure chemotherapy. Br J Radiol.

[CR5] Benson AB, Bekaii-Saab T, Chan E (2013). Metastatic colon cancer, version 3.2013: featured updates to the NCCN guidelines. J Natl Comprehen Cancer Netw.

[CR6] de Baere T, Roche A, Vavasseur D, Therasse E, Indushekar S, Elias D, Bognel C (1993). Portal vein embolization: utility for inducing left hepatic lobe hypertrophy before surgery. Radiology.

[CR7] de Meijer VE, Kalish BT, Puder M, Ijzermans JN (2010). Systematic review and meta-analysis of steatosis as a risk factor in major hepatic resection. Br J Surg.

[CR8] Deipolyi AR, Zhang YS, Khademhosseini A, Naidu S, Borad M, Sahin B, Mathur A, Oklu R (2017). Portal vein embolization: impact of chemotherapy and genetic mutations. J Clin Med.

[CR9] Dindo D, Demartines N, Clavien P-A (2004). Classification of surgical complications: a new proposal with evaluation in a cohort of 6336 patients and results of a survey. Ann Surg.

[CR10] Ebata T, Yokoyama Y, Igami T, Sugawara G, Takahashi Y, Nagino M (2012). Portal vein embolization before extended hepatectomy for biliary cancer: current technique and review of 494 consecutive embolizations. Dig Surg.

[CR11] Filippiadis DK, Binkert C, Pellerin O, Hoffmann RT, Krajina A, Pereira PL (2017). Cirse quality assurance document and standards for classification of complications: the Cirse classification system. Cardiovasc Intervent Radiol.

[CR12] Geisel D, Malinowski M, Powerski MJ, Wüstefeld J, Heller V, Denecke T, Stockmann M, Gebauer B (2014). Improved hypertrophy of future remnant liver after portal vein embolization with plugs, coils and particles. Cardiovasc Intervent Radiol.

[CR13] Guiu B, Quenet F, Escal L, Bibeau F, Piron L, Rouanet P, Fabre JM, Jacquet E, Denys A, Kotzki PO, Verzilli D, Deshayes E (2017). Extended liver venous deprivation before major hepatectomy induces marked and very rapid increase in future liver remnant function. Eur Radiol.

[CR14] Hahn O, Bárdos D, Kupcsulik P, Szijártó A, Fülöp A, Kokas B, Pekli D, Zsirka-Klein A, Dudás I, Pajor P, Harsányi L (2019). Decreasing morbidity after associating liver partition and portal vein ligation for staged hepatectomy (ALPPS) with technical modification and patient selection. Orv Hetil.

[CR15] Hwang S, Ha TY, Ko GY, Kwon DI, Song GW, Jung DH, Kim MH, Lee SK, Lee SG (2015). Preoperative sequential portal and hepatic vein embolization in patients with Hepatobiliary malignancy. World J Surg.

[CR16] Jaberi A, Toor SS, Rajan DK (2016). Comparison of clinical outcomes following glue versus polyvinyl alcohol portal vein embolization for hypertrophy of the future liver remnant prior to right hepatectomy. J Vasc intervent Radiol.

[CR17] Kambakamba P, Stocker D, Reiner CS, Nguyen-Kim TD, Linecker M, Eshmuminov D, Petrowsky H, Clavien PA, Lesurtel M (2016). Liver kinetic growth rate predicts postoperative liver failure after ALPPS. HPB.

[CR18] Kinoshita Y, Nonaka H, Suzuki S (1985). Accurate localization of insulinoma using percutaneous transhepatic portal venous sampling--usefulness of simultaneous measurement of plasma insulin and glucagon levels. Clin Endocrinol.

[CR19] Kubota K, Makuuchi M, Kusaka K (1997). Measurement of liver volume and hepatic functional reserve as a guide to decision-making in resectional surgery for hepatic tumors. Hepatology (Baltimore, Md.).

[CR20] Le Roy B, Perrey A, Fontarensky M (2017). Combined preoperative portal and hepatic vein embolization (Biembolization) to improve liver regeneration before major liver resection: a preliminary report. World J Surg.

[CR21] Lee EC, Park S-J, Han S-S, Park HM, Lee SD, Kim SH, Lee IJ, Kim HB (2017). Mortality after portal vein embolization: two case reports. Medicine (Baltimore).

[CR22] Linecker M, Björnsson B, Stavrou GA, Oldhafer KJ, Lurje G, Neumann U, Adam R, Pruvot FR, Topp SA, Li J, Capobianco I, Nadalin S, Machado MA, Voskanyan S, Balci D, Hernandez-Alejandro R, Alvarez FA, de Santibañes E, Robles-Campos R, Malagó M, de Oliveira ML, Lesurtel M, Clavien PA, Petrowsky H (2017). Risk adjustment in ALPPS is associated with a dramatic decrease in early mortality and morbidity. Ann Surg.

[CR23] Luz JHM, Gomes FV, Coimbra E, Costa NV, Bilhim T (2020). Preoperative portal vein embolization in hepatic surgery: a review about the embolic materials and their effects on liver regeneration and outcome. Radiol Res Pract.

[CR24] Madoff DC, Abdalla EK, Gupta S, Wu TT, Morris JS, Denys A, Wallace MJ, Morello FA, Ahrar K, Murthy R, Lunagomez S, Hicks ME, Vauthey JN (2005). Transhepatic ipsilateral right portal vein embolization extended to segment IV: improving hypertrophy and resection outcomes with spherical particles and coils. J Vasc Intervent Radiol.

[CR25] Madoff DC, Gaba RC, Weber CN, Clark TW, Saad WE (2016). Portal venous interventions: state of the art. Radiology.

[CR26] Makuuchi M, Thai BL, Takayasu K, Takayama T, Kosuge T, Gunvén P, Yamazaki S, Hasegawa H, Ozaki H (1990). Preoperative portal embolization to increase safety of major hepatectomy for hilar bile duct carcinoma: a preliminary report. Surgery.

[CR27] Nagino M, Kamiya J, Nishio H, Ebata T, Arai T, Nimura Y (2006). Two hundred forty consecutive portal vein embolizations before extended hepatectomy for biliary cancer: surgical outcome and long-term follow-up. Ann Surg.

[CR28] Narita M, Oussoultzoglou E, Jaeck D, Fuchschuber P, Rosso E, Pessaux P, Marzano E, Bachellier P (2011). Two-stage hepatectomy for multiple bilobar colorectal liver metastases. Br J Surg.

[CR29] Ribero D, Abdalla EK, Madoff DC, Donadon M, Loyer EM, Vauthey JN (2007). Portal vein embolization before major hepatectomy and its effects on regeneration, resectability and outcome. Br J Surg.

[CR30] Sakuhara Y, Abo D, Hasegawa Y, Shimizu T, Kamiyama T, Hirano S, Fukumori D, Kawamura T, Ito YM, Tha KK, Shirato H, Terae S (2012). Preoperative percutaneous transhepatic portal vein embolization with ethanol injection. AJR Am J Roentgenol.

[CR31] Schnitzbauer AA, Lang SA, Goessmann H, Nadalin S, Baumgart J, Farkas SA, Fichtner-Feigl S, Lorf T, Goralcyk A, Hörbelt R, Kroemer A, Loss M, Rümmele P, Scherer MN, Padberg W, Königsrainer A, Lang H, Obed A, Schlitt HJ (2012). Right portal vein ligation combined with in situ splitting induces rapid left lateral liver lobe hypertrophy enabling 2-staged extended right hepatic resection in small-for-size settings. Ann Surg.

[CR32] Shindoh J, Truty MJ, Aloia TA, Curley SA, Zimmitti G, Huang SY, Mahvash A, Gupta S, Wallace MJ, Vauthey JN (2013). Kinetic growth rate after portal vein embolization predicts posthepatectomy outcomes: toward zero liver-related mortality in patients with colorectal liver metastases and small future liver remnant. J Am Coll Surg.

[CR33] Shirabe K, Shimada M, Gion T, Hasegawa H, Takenaka K, Utsunomiya T, Sugimachi K (1999). Postoperative liver failure after major hepatic resection for hepatocellular carcinoma in the modern era with special reference to remnant liver volume. J Am Coll Surg.

[CR34] Shoup M, Gonen M, D'Angelica M, Jarnagin WR, DeMatteo R, Schwartz LH, Tuorto S, Blumgart LH, Fong Y (2003). Volumetric analysis predicts hepatic dysfunction in patients undergoing major liver resection. J Gastrointest Surg.

[CR35] Takayasu K, Muramatsu Y, Shima Y, Moriyama N, Yamada T, Makuuchi M (1986). Hepatic lobar atrophy following obstruction of the ipsilateral portal vein from hilar cholangiocarcinoma. Radiology.

[CR36] Tanaka H, Kinoshita H, Hirohashi K, Kubo S, Lee KC (1994). Increased safety by two-stage hepatectomy with preoperative portal vein embolization in rats. J Surg Res.

[CR37] Tustumi F, Ernani L, Coelho FF, Bernardo WM, Junior SS, Kruger JAP, Fonseca GM, Jeismann VB, Cecconello I, Herman P (2018). Preoperative strategies to improve resectability for hepatocellular carcinoma: a systematic review and meta-analysis. HPB.

[CR38] van Lienden KP, van den Esschert JW, de Graaf W, Bipat S, Lameris JS, van Gulik TM, van Delden OM (2013). Portal vein embolization before liver resection: a systematic review. Cardiovasc Intervent Radiol.

[CR39] Vauthey JN, Chaoui A, Do KA, Bilimoria MM, Fenstermacher MJ, Charnsangavej C, Hicks M, Alsfasser G, Lauwers G, Hawkins IF, Caridi J (2000). Standardized measurement of the future liver remnant prior to extended liver resection: methodology and clinical associations. Surgery.

[CR40] Wajswol E, Jazmati T, Contractor S, Kumar A (2018). Portal vein embolization utilizing N-butyl cyanoacrylate for contralateral lobe hypertrophy prior to liver resection: a systematic review and meta-analysis. Cardiovasc Intervent Radiol.

[CR41] Wanis KN, Linecker M, Madenci AL, Müller PC, Nüssler N, Brusadin R, Robles-Campos R, Hahn O, Serenari M, Jovine E, Lehwald N, Knoefel WT, Reese T, Oldhafer K, de Santibañes M, Ardiles V, Lurje G, Capelli R, Enne M, Ratti F, Aldrighetti L, Zhurbin AS, Voskanyan S, Machado M, Kitano Y, Adam R, Chardarov N, Skipenko O, Ferri V, Vicente E, Tomiyama K, Hernandez-Alejandro R (2021). Variation in complications and mortality following ALPPS at early-adopting centers. HPB.

[CR42] Zeile M, Bakal A, Volkmer JE, Stavrou GA, Dautel P, Hoeltje J, Stang A, Oldhafer KJ, Brüning R (2016). Identification of cofactors influencing hypertrophy of the future liver remnant after portal vein embolization-the effect of collaterals on embolized liver volume. Br J Radiol.

[CR43] Zorzi D, Chun YS, Madoff DC, Abdalla EK, Vauthey JN (2008). Chemotherapy with bevacizumab does not affect liver regeneration after portal vein embolization in the treatment of colorectal liver metastases. Ann Surg Oncol.

